# Phytochemical Modulation of the Kynurenine Pathway (KYNP) and Its Emerging Mechanistic Insights into Cancer Progression: A Systematic Review

**DOI:** 10.3390/ijms27177512

**Published:** 2026-08-22

**Authors:** Evgenia Maria Tsantila, Marios C. Christodoulou, Nils Esslinger, Christiana M. Neophytou

**Affiliations:** 1 Department of Life Sciences, European University Cyprus, Nicosia 2404, Cyprus; et221233@students.euc.ac.cy (E.M.T.); marioschemist@outlook.com (M.C.C.); 2 Department of Research and Development, Alpinamed AG, 9306 Freidorf, Switzerland; nils.esslinger@alpinamed.ch

**Keywords:** kynurenine pathway, phytochemicals, tryptophan metabolism, IDO1, IDO2, TDO2

## Abstract

Dysregulation of the kynurenine pathway (KYNP) is increasingly recognized as a hallmark of cancer-associated metabolic reprogramming, contributing to immune evasion, oxidative stress, and tumor progression through the activity of enzymes such as indoleamine 2,3-dioxygenase 1 (IDO1), indoleamine 2,3-dioxygenase 2 (IDO2), and tryptophan 2,3-dioxygenase (TDO2). This systematic review aimed to evaluate the current evidence on the ability of phytochemicals to modulate the KYNP and their potential implications for cancer prevention and therapy. The review was conducted in accordance with the Preferred Reporting Items for Systematic Reviews and Meta-Analyses (PRISMA) guidelines and included English-language articles and book chapters published between 2016 and 2026 that were retrieved from the Scopus and PubMed databases. A keyword co-occurrence network was generated using VOSviewer (v1.6.20) based on the complete Scopus and PubMed exports of the included studies to identify major research themes. The available evidence indicates that phytochemicals restore anticancer immunity by targeting multiple components of the KYNP, including inhibition of IDO1-mediated kynurenine production and suppression of downstream aryl hydrocarbon receptor signalling, thereby enhancing cytotoxic T-cell responses, reducing immunosuppressive cell populations, and improving antitumor immune activity. Collectively, these findings support the KYNP as a promising immunometabolic target and highlight phytochemicals as potential complementary agents for cancer immunotherapy while emphasizing the need for further investigation of the biological and immunological roles of IDO2.

## 1. Introduction

Phytochemicals are naturally occurring compounds found in plants, where they perform diverse ecological and protective functions. Owing to their considerable chemical diversity, they can be broadly classified according to their chemical structures and functional groups into several major categories, including alkaloids, polyphenols, flavonoids, saponins, terpenes, and carotenoids [1]. In their native environment, these compounds help plants defend against pests, protect their tissues from damage caused by ultraviolet (UV) radiation, and enhance their resistance to pathogens and environmental stressors [2]. When consumed as part of the human diet, phytochemicals may also exert beneficial biological effects by modulating antioxidant defense mechanisms, inflammatory responses, and physiological pathways associated with chronic disease development [3,4].

Among their most extensively studied biological properties is their ability to scavenge free radicals. For example, flavonoids such as quercetin, catechin, and epigallocatechin gallate (EGCG) can directly neutralize reactive oxygen species (ROS) and inhibit lipid peroxidation [5,6]. Phenolic acids, including caffeic and ferulic acid, also exhibit strong antioxidant activity by donating hydrogen atoms and stabilizing free radicals [7]. Similarly, carotenoids, such as β-carotene, lycopene, and lutein, reduce oxidative damage to lipids, proteins, and DNA [8]. Beyond their antioxidant activity, the therapeutic relevance of plant-derived compounds in oncology is demonstrated by the established clinical use of paclitaxel, vincristine, and camptothecin derivatives as anticancer agents [9]. Their multipurpose characteristics highlight their value as both preventive agents and potential candidates for synergistic cancer treatment strategies [10]. Table 1 presents well-established phytochemicals and their biological activity.

Moreover, increasing emphasis has been placed on their potential ability to regulate metabolic and immune signaling pathways involved in cancer progression. Recent research indicates that these substances may modify the kynurenine pathway (KYNP) by influencing the expression and activity of its essential enzymes, most notably indoleamine 2,3-dioxygenase 1 (IDO1), as well as related signaling pathways involved in oxidative stress and inflammation [11].

In particular, the KYNP is the major route of tryptophan (Trp) catabolism and is responsible for the metabolism of approximately 95% of available Trp [12]. Trp is an essential amino acid that serves not only as a building block for protein synthesis but also as a precursor for several biologically important metabolites. Although smaller proportions of Trp are used for serotonin and melatonin biosynthesis or are metabolized by the gut microbiota into indole derivatives [12,13], most Trp is converted through the KYNP into kynurenine (Kyn) and several biologically active downstream metabolites. The initial and rate-limiting step of the KYNP is catalyzed by three dioxygenases: IDO1, indoleamine 2,3-dioxygenase 2 (IDO2), and tryptophan 2,3-dioxygenase (TDO2). These enzymes catalyze the oxidative cleavage of the indole ring of Trp to produce N-formyl-L-kynurenine, which is subsequently converted into Kyn [14,15]. Kyn can then be metabolized through different enzymatic branches to generate several bioactive metabolites, including kynurenic acid (KYNA), 3-hydroxykynurenine (3-HK), xanthurenic acid (XA), 3-hydroxyanthranilic acid (3-HAA), and quinolinic acid (QUIN), ultimately contributing to de novo nicotinamide adenine dinucleotide (NAD^+^) biosynthesis [16,17,18]. These metabolites exert diverse and sometimes opposing effects on immune regulation, oxidative stress, and cellular homeostasis.

The three rate-limiting enzymes differ in their tissue distribution, catalytic activity, and regulatory mechanisms. TDO2 is predominantly expressed in the liver, where it contributes to systemic Trp homeostasis and is regulated by factors such as Trp availability and glucocorticoids [16,19]. In contrast, IDO1 is expressed in several extrahepatic tissues and immune-cell populations and is strongly induced by inflammatory mediators, particularly interferon-γ (IFN-γ) [20]. IDO2 exhibits lower catalytic activity and a more restricted expression pattern than IDO1, and its biological and immunological functions remain less clearly understood [21]. Collectively, these enzymes regulate Trp availability and the production of KYNP metabolites, thereby influencing metabolic homeostasis and immune-cell activity.

Aberrant activation of the KYNP has been reported in numerous tumor types and is increasingly recognized as an important mechanism of tumor-associated immune suppression. Upregulation of IDO1, IDO2, or TDO2 in tumor cells and tumor-associated immune cells may result in local Trp depletion and the accumulation of immunoregulatory metabolites, particularly Kyn. This metabolic reprogramming suppresses effector T-cell proliferation, promotes the differentiation of regulatory T cells (Tregs), and contributes to the establishment of an immunosuppressive tumor microenvironment (TME), thereby facilitating immune evasion and tumor progression [22]. Kyn and certain downstream metabolites can also activate the aryl hydrocarbon receptor (AhR), which regulates genes involved in immune function, cellular metabolism, and angiogenesis [23]. Activation of the IDO1–Kyn–AhR axis has additionally been associated with increased programmed cell death protein 1 (PD-1) expression on CD8^+^ T cells, further promoting immune tolerance within the TME [24]. Other KYNP metabolites, including 3-HK and QUIN, may contribute to oxidative stress, redox imbalance, and DNA damage, illustrating the complex involvement of the pathway in cancer biology [25].

Despite growing interest in the immunometabolic functions of the KYNP, evidence regarding its modulation by phytochemicals remains fragmented. In particular, the effects of phytochemicals on individual KYNP enzymes and downstream metabolites have not been comprehensively synthesized, while the catalytic and immunological functions of IDO2 remain comparatively understudied. This systematic review therefore aims to identify, evaluate, and integrate the available evidence regarding phytochemical-mediated modulation of the KYNP and its potential implications for cancer prevention and therapy. Particular attention is given to the effects of phytochemicals on IDO1, IDO2, and TDO2; the production and activity of downstream metabolites; and the associated regulation of oxidative stress, immune tolerance, and TME. By addressing these areas, this review seeks to clarify current evidence, identify important knowledge gaps, and establish priorities for future mechanistic, preclinical, and clinical research.

## 2. Materials and Methods

### 2.1. PRISMA Flowchart

Using the PRISMA statement (Preferred Reporting Items for Systematic Reviews and Meta-Analysis), a collection solely of articles and book chapters was chosen for the present review, by counting only those written in the English language [26]. For the current purpose, the Scopus and PubMed databases were used to include all the studies from 2016 to 2026. The search strategy employed the following keywords:

“phytochemical” OR “plant-derived compound” OR “flavonoid” OR “polyphenol” OR “alkaloid” OR “terpenoid” OR “phenolic” AND “kynurenine” OR “kynurenine pathway” OR “kynurenic” OR “kynurenic acid” OR “quinolinic acid” OR “3-hydroxykynurenine” AND “cancer” OR “carcinoma” OR “neoplasm” OR “tumor” OR “tumour” OR “malignant” OR “oncology”. This comprehensive search retrieved 155 publications, from Scopus and PubMed. To improve the specificity and quality of the dataset, results were further refined through the application of predefined inclusion and exclusion criteria, as detailed in Table 2.

Afterwards, titles and abstracts were manually screened to ensure that each study was:

1. Investigated plant-derived compounds (phytochemicals)
2. specifically assessed their impact on the KYNP or its metabolites, and
3. demonstrated relevance to cancer mechanisms.

Duplicates were removed using Mendeley software version 2.148.0. Studies were excluded if they lacked mechanistic data linking phytochemicals to KYNP, focused on non-cancer diseases, or were unavailable in full text. Following both automated and manual screening, only 17 publications met the inclusion criteria.

The final literature search was conducted on 11 June 2026. Studies available in PubMed and Scopus by the date of the final search were considered for eligibility according to the predefined inclusion and exclusion criteria, including articles available online ahead of print when indexed in the searched databases. All 2026 publications included in the present review were already available as published articles by the date of the final search.

### 2.2. Methodological Quality and Risk-of-Bias Assessment

Methodological quality was appraised at the level of each relevant experimental component using design-specific tools. Animal intervention experiments were evaluated with SYRCLE’s 10-domain risk-of-bias tool [27], covering sequence generation, baseline characteristics, allocation concealment, random housing, blinding of investigators, random outcome assessment, blinded outcome assessment, incomplete outcome data, selective reporting, and other bias. Each domain was judged low, high, or unclear risk; no overall numerical score was calculated.

In vitro, cell-free, and ex vivo components were evaluated using SciRAP version 2.0 [28]. Reporting- and methodological-quality criteria were rated fulfilled, partially fulfilled, not fulfilled, not determined, or not applicable and summarized across test-compound/control, test-system, exposure, data-collection/analysis, and funding/competing-interest domains. Relevance to the review question was rated direct, indirect, not relevant, or not determined.

The computational-only study was evaluated with a prespecified checklist based on reliability and reproducibility guidance for molecular simulations [29], addressing structure and ligand provenance, software and parameters, controls and validation, convergence, input/code availability, and connection to experimental evidence. Appraisals used the complete articles and supporting files for all 17 studies. Quality judgments were used to qualify the narrative synthesis rather than to exclude studies or generate a pooled score.

### 2.3. VOSviewer

A keyword co-occurrence network generated by VOSviewer (v1.6.20) from the complete Scopus CSV export of 17 articles was also conducted to map research themes. The analysis relied on author keywords and employed the full-counting method. Imposing a minimum occurrence threshold of two, retained 27 of the 27 unique keywords for mapping. VOSviewer’s clustering algorithm then partitioned these terms into discrete thematic clusters, each rendered in a distinct colour. In the resulting network visualization node size is proportional to keyword frequency, and link thickness reflects co-occurrence strength. In addition, the percentage of studies investigating each biological matrix across the 155 papers was calculated. Finally, two complementary visualizations were produced: a network diagram and a density or heatmap diagram, each offering unique insights into the structure and focus of the research landscape.

## 3. Results

### 3.1. Literature Screening and Selection

**Figure 1:**
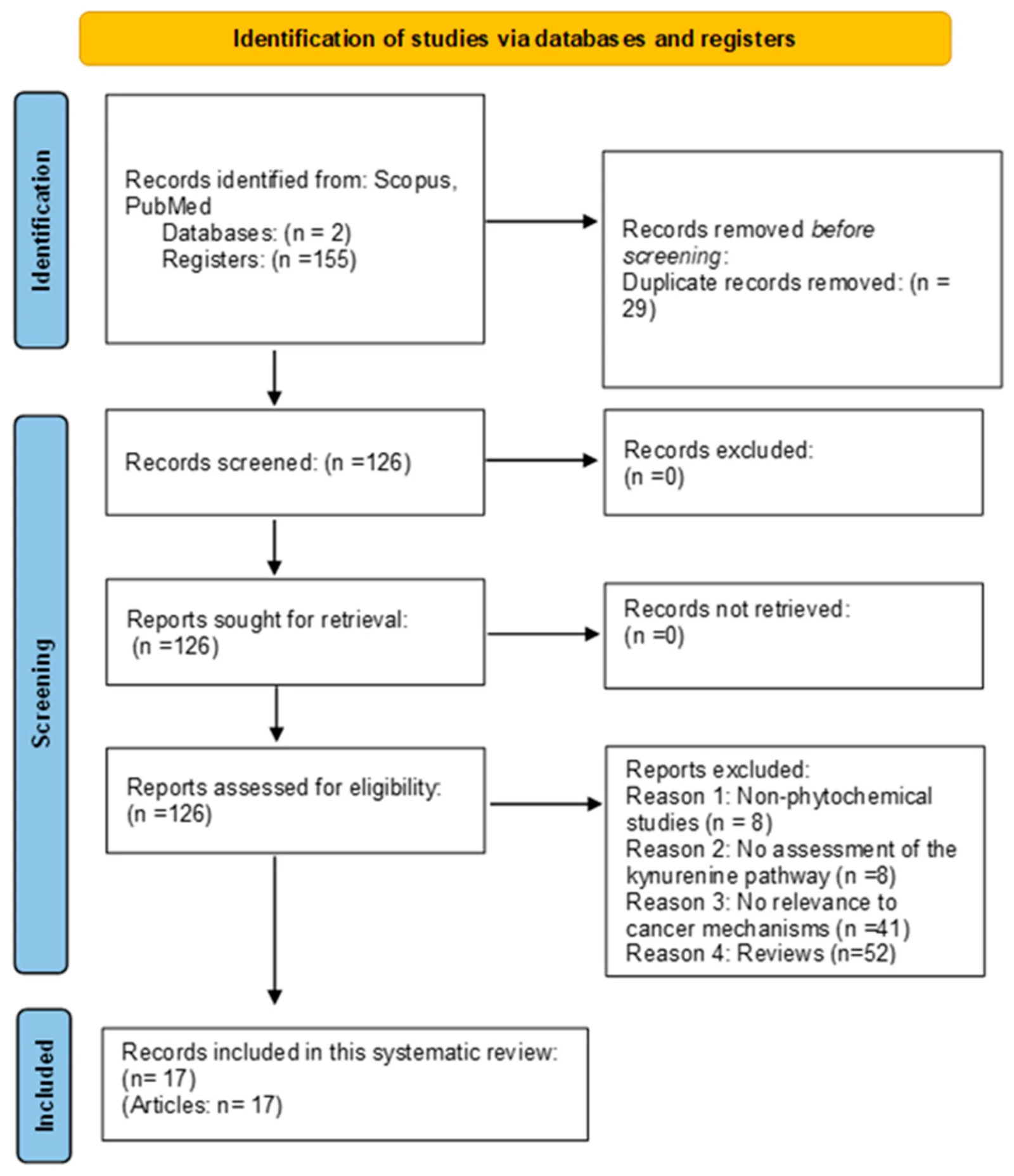
PRISMA flow diagram.

A total of 155 records were identified through searches of the Scopus and PubMed databases. After the removal of 29 duplicate records, 126 records remained and were screened. No records were excluded during the title and abstract screening stage, and all 126 reports were sought for retrieval and assessed for eligibility. Following full-text assessment, 109 reports were excluded for the following reasons: non-phytochemical studies (*n* = 8), no assessment of the kynurenine pathway (*n* = 8), no relevance to cancer mechanisms (*n* = 41), and review articles (*n* = 52). Consequently, 17 original research articles published between 2016 and 2026 met the eligibility criteria and were included in the final systematic review (Figure 1).

### 3.2. Keyword Co-Occurrence Network Analysis

**Figure 2:**
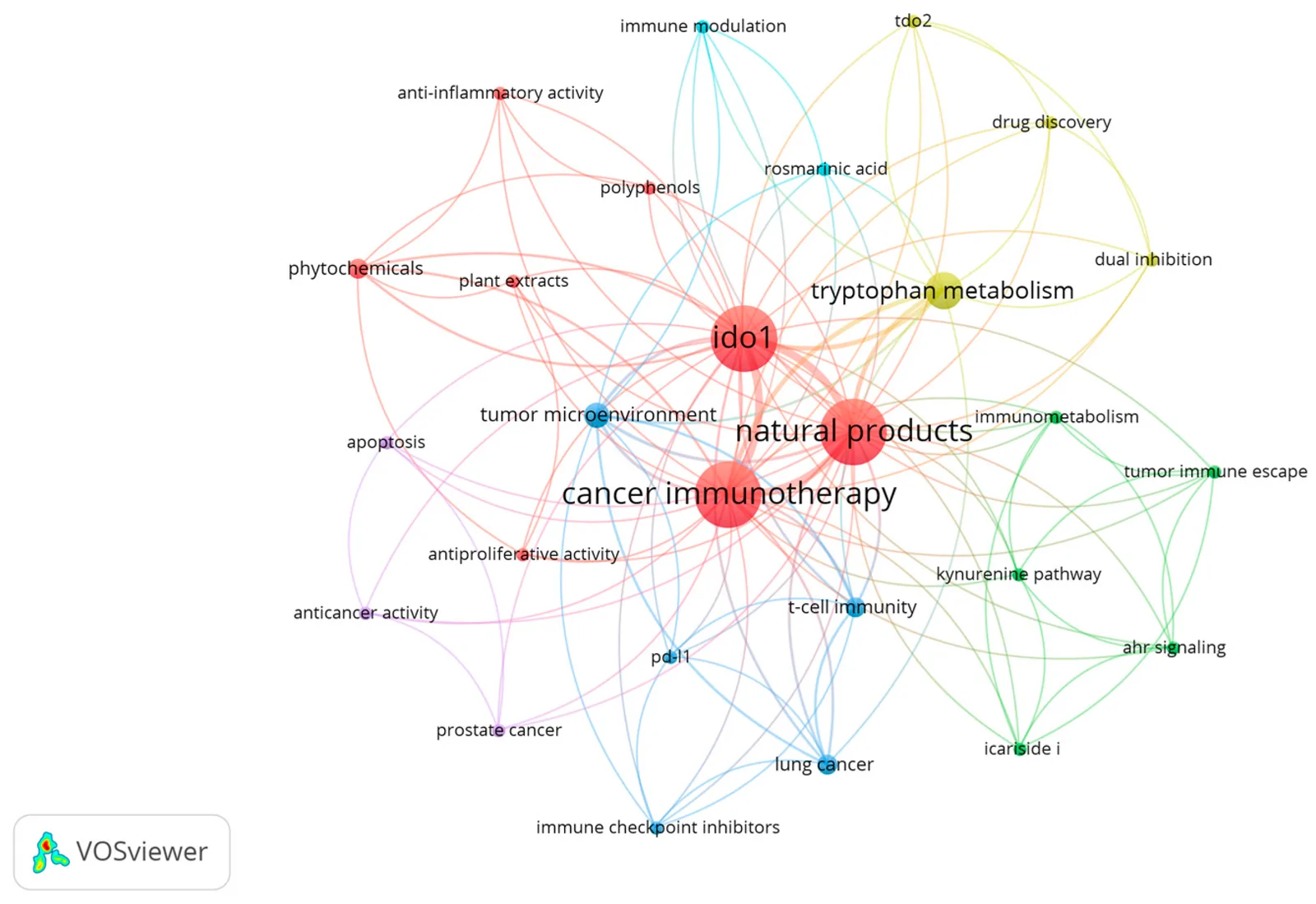
Keyword co-occurrence network map of the 17 studies included in the systematic review, generated using VOSviewer software version 1.6.20. Each node represents a keyword, and the size of the node reflects its frequency of occurrence within the analyzed publications. The connecting lines indicate co-occurrence relationships between terms, while different colors represent distinct thematic clusters.

Figure 2 presents the keyword co-occurrence network map generated from the 17 studies included in this systematic review, illustrating the major thematic relationships within the literature on phytochemical modulation of the KYNP in cancer. The analysis identified four principal clusters, each representing a distinct but interconnected research focus. The first cluster (red) is centered around terms such as IDO1, natural products, and cancer immunotherapy, highlighting the primary focus of the included studies on the development of natural product-based strategies to modulate immune responses in cancer. This cluster also includes terms such as phytochemicals, polyphenols, and plant extracts, reflecting the growing interest in bioactive compounds as potential immunotherapeutic agents. The second cluster (green), comprising keywords including kynurenine pathway, AhR signaling, tumor immune escape, Icariside-I, and immunometabolism, represents studies investigating the downstream immunological consequences of Trp metabolism and the role of KYNP-derived metabolites in promoting immune evasion. The third cluster (blue), which includes tryptophan metabolism, T-cell immunity, PD-1, and immune checkpoint inhibitors, emphasizes the relationship between IDO1 activity and antitumor immune responses. The yellow cluster, characterized by TDO2, drug discovery, and dual inhibition, reflects research focused on the enzymatic regulation of Trp metabolism and the identification of novel therapeutic targets within the KYNP. In addition, a smaller purple cluster is characterized by terms such as apoptosis, antiproliferative activity, anticancer activity, and prostate cancer, reflecting research focused on the downstream antitumor effects associated with phytochemical-mediated KYNP modulation. A further light blue cluster includes immune modulation as a prominent term, highlighting the broader immunoregulatory effects associated with phytochemical activity and KYNP-related signaling. Collectively, these clusters reveal a well-integrated research landscape in which Kyn serves as a central hub linking enzymatic regulation, immunometabolic responses, and phytochemical-mediated therapeutic interventions in cancer.

**Figure 3:**
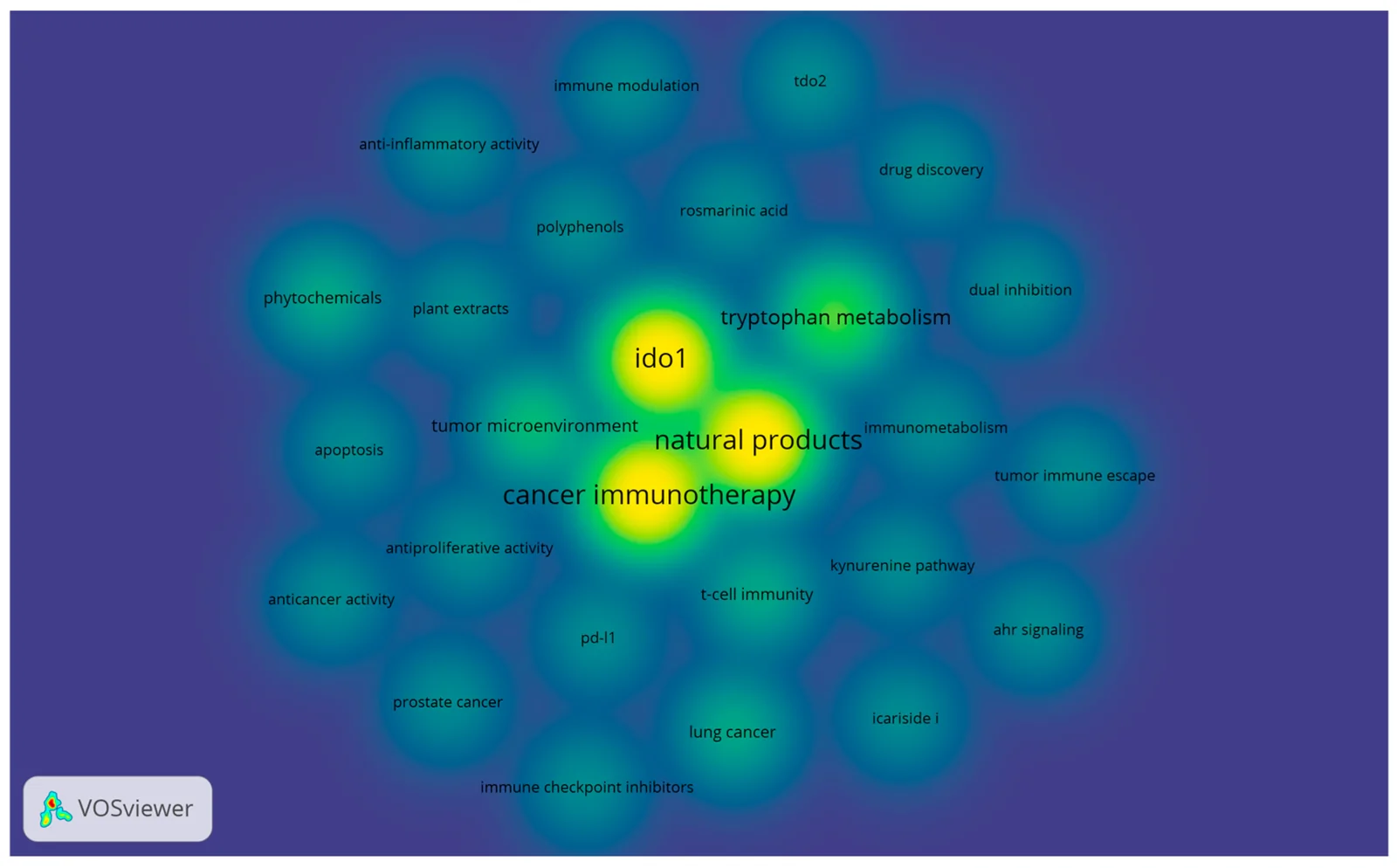
Keyword co-occurrence network heatmap of the 17 studies included in the systematic review, generated using VOSviewersoftware version 1.6.20. The size of each term reflects its frequency of occurrence, while color intensity indicates the density of related research topics. Central clusters highlight strong associations between natural products, IDO1, and cancer-related studies.

The heatmap in Figure 3 also clearly indicates that research on phytochemical modulation of the KYNP in cancer exhibits a multidimensional, interconnected structure encompassing enzymatic regulation, immune modulation, and natural compound–based therapeutic strategies. The heatmap identifies IDO1, natural products, and cancer immunotherapy as the most influential and interconnected keywords in the network. The distinction of Trp metabolism further highlights the importance of KYNP regulation in cancer. The co-occurrence of these terms with phytochemicals, polyphenols, and plant extracts reflects growing interest in natural compounds as modulators of the IDO1-mediated immunosuppressive pathways and potential enhancers of cancer immunotherapy.

### 3.3. Temporal Trends

**Figure 4:**
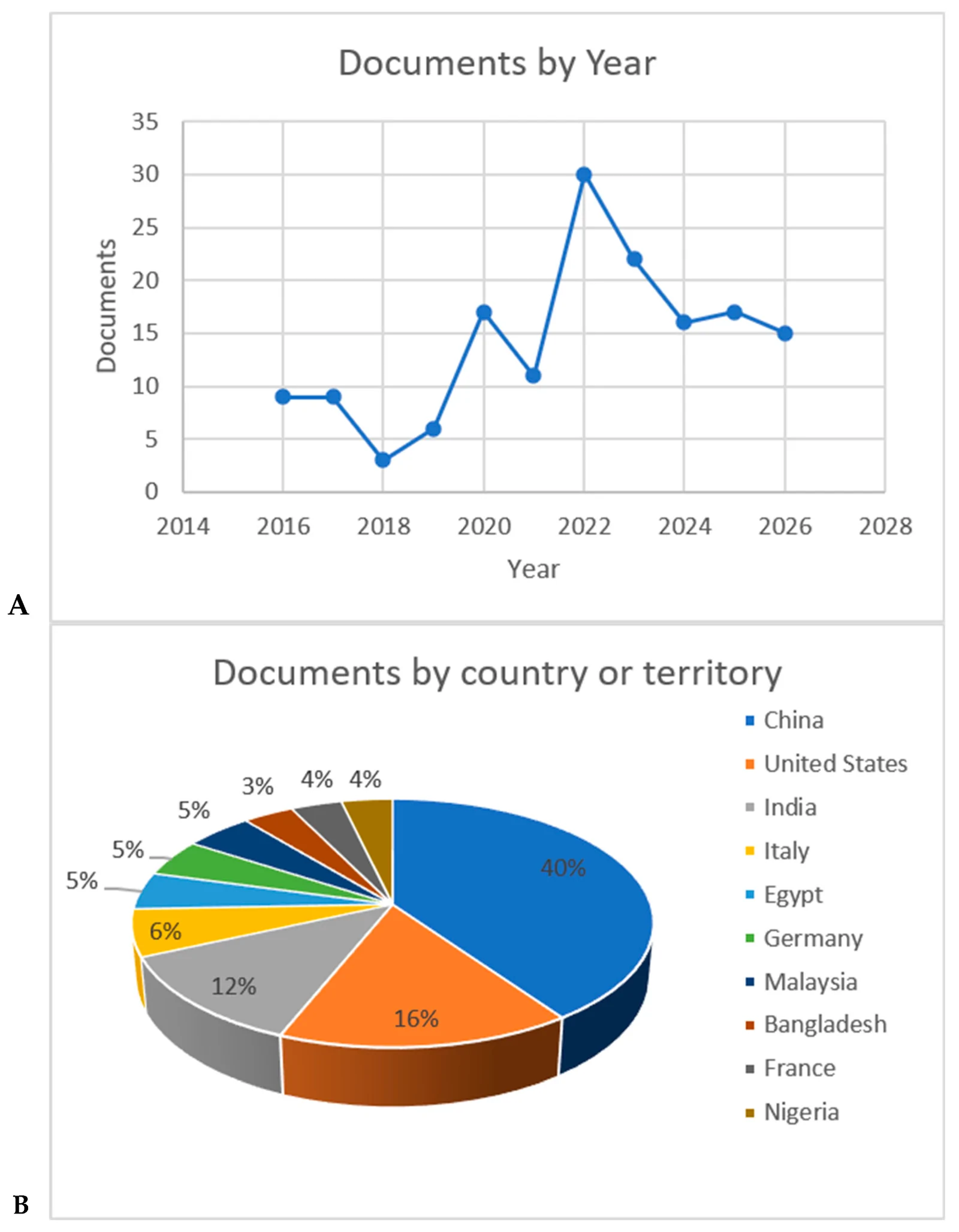
Temporal evolution and global distribution of manuscripts on phytochemical modulation of the KYNP in cancer. (**A**). Number of documents published each year (2016–2026) based on research on Scopus and PubMed data. The trend shows a significant increase in publications beginning in 2019 and peaking between 2021 and 2024, demonstrating an increasing interest in the interaction of phytochemicals and the kynurenine pathway in cancer research. (**B**). Number of publications by country related to phytochemicals and the kynurenine pathway in cancer. China has the highest research output, followed by the United States, India, and Italy. Bangladesh, Egypt, Germany, Malaysia, France, and Nigeria are also making new contributions, indicating a growing international interest in this area. Data were obtained from the Scopus database and PubMed and visualized using Microsoft Excel.

This section examines the temporal evolution and global distribution of research on phytochemical modulation of the KYNP in cancer. Figure 4A,B present an overview of the bibliometric characteristics of the literature examined in this study. Specifically, Figure 4A illustrates the annual distribution of publications from 2016 to 2026, demonstrating how scientific output has changed over time and showing a noticeable rise in research effort in recent years. This trend reflects the growing academic interest and expanding investigation of the interaction between phytochemicals and KYNP. Additionally, the worldwide contribution to this topic is illustrated by Figure 4B, which shows the geographical distribution of publications by country. According to the data, China is the country that provides the most research, followed by the US, India, and Italy. When evaluated as a whole, these numbers offer a quantitative basis for understanding the evolution of this field’s research over time as well as its global reach.

### 3.4. Characteristics of the Included Studies

A total of 17 studies met the predefined eligibility criteria and were included in this systematic review. The included studies investigated a wide range of naturally derived compounds, including flavonoids, polyphenols, anthraquinones, alkaloids, terpenoid derivatives, herbal extracts and phytochemical-based formulations, demonstrating the increasing interest in natural compounds as KYNP modulators. The majority of studies used in vitro assays, but some also used in vivo models, or the two approaches were combined. In addition, a limited number of studies used in silico molecular docking or enzyme-based assays to identify novel inhibitors of key KYNP enzymes. Despite significant differences in their chemical structures and experimental approaches, the phytochemicals under investigation affected similar molecular processes. Most of the studies focused on the inhibition of IDO1, whereas some investigated dual targeting of IDO1 and TDO2 or modulation of downstream Kyn metabolites. Beyond the direct enzyme inhibition, numerous studies demonstrated that phytochemicals regulate multiple signaling pathways associated with cancer progression, including PI3K/AKT, STAT3, NF-κB, oxidative stress-related pathways, and immune regulatory networks. The data were synthesized based on their major biological impacts, rather than by phytochemical class, due to the mechanistic diversity of the included investigations. As a result, the following sections summarize the available evidence on phytochemical-mediated regulation of the KYNP, focusing on (i) direct modulation of KYNP entry enzymes (IDO1 and TDO2), (ii) regulation of Kyn metabolism and downstream metabolites, (iii) cellular consequences associated with phytochemical-mediated KYNP modulation in cancer, and (iv) KYNP-driven immunomodulation and TME remodeling. Finally, emerging mechanistic patterns and future therapeutic perspectives were identified across the included studies. Table 3 summarizes the basic characteristics of the reviewed articles.

### 3.5. Methodological Quality and Risk of Bias

Seven studies contained animal intervention experiments [32,35,36,41,42,45,46]. Reporting of safeguards against bias was limited. Random allocation was stated in four studies [32,35,36,45], but none described the sequence-generation method. High risk for incomplete outcome data was assigned to [35,45] because allocated group sizes differed from several outcome-specific sample sizes without explanation. Study [41] was judged at high risk for other bias because the reported tumour-implantation route was internally inconsistent and analyses across several groups relied on multiple pairwise tests. In ref. [46], randomization was stated without a sequence method, and immune outcomes used five animals from groups of seven without a reported selection or attrition rationale.

Across 15 studies with in vitro, enzyme, or ex vivo components [30,31,33,35,36,37,38,39,40,41,42,43,44,45,46], test systems, exposures, and measured outcomes were usually described, but recurrent limitations included incomplete compound characterization, limited reporting of independent biological replication, absent sample-size justification, and incomplete multiplicity or post-hoc procedures. Confidence was comparatively stronger for the replicated, orthogonal experiments in [38,43]. Study [31] provided only indirect cancer relevance, and internal inconsistencies in study [41] materially reduced confidence. These judgments were used to qualify the synthesis; they were not converted into a pooled numerical score.

The full computational-only study [34] reported protein structures, a 325-compound screening library, software versions, reference-ligand redocking, and 150-ns molecular-dynamics settings. Reproducibility nevertheless remained limited by incomplete identification of screening inputs and parameters, absence of independent trajectories or formal convergence testing, and lack of reusable input files, code, and experimental validation. Table 4 provides the study-level confidence synthesis. Complete domain-level judgments and rationales are reported in Supplementary Tables S2–S4.

**Figure 5:**
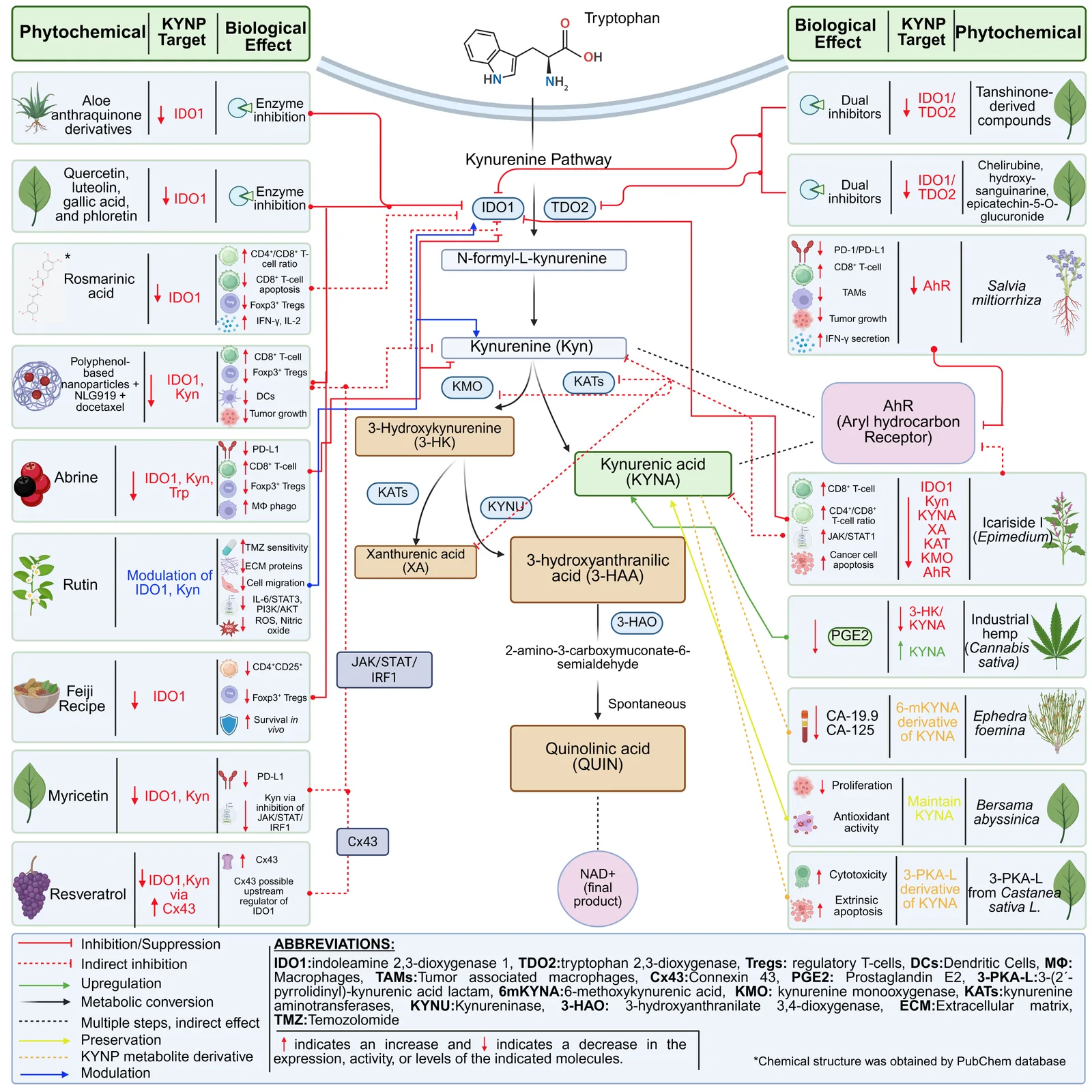
Graphical summary of the reported actions of various phytochemicals on the KYNP, and their immunological and biological consequences in cancer, based on the included studies. Created in BioRender. Gkretsi, V. (2026) https://BioRender.com/s5ibnee.

Figure 5 summarizes the major findings regarding bioactive phytochemicals, their effect on the KYNP and its biological output. Specifically, it illustrates the principal molecular targets of the selected phytochemicals within the KYNP, including the modulation of IDO1, TDO2, AhR signalling, and downstream Kyn metabolites. The figure highlights the associated biological outcomes, such as immune regulation, suppression of tumor growth, enhancement of antitumor immunity, and induction of apoptosis, providing an integrated overview of the mechanisms through which phytochemicals may exert anticancer effects by targeting the KYNP.

## 4. Discussion

Given its central role in immune and metabolic regulation, KYNP has emerged as a key target of interest in phytochemical research. Among the various molecular mechanisms through which phytochemicals exert their biological effects, increasing attention has been directed toward their ability to modulate Trp metabolism, particularly through the regulation of KYNP enzymes and downstream metabolites [47].

The first and rate-limiting step of the KYNP which is the conversion of Trp to N-formyl-L-kynurenine, is catalyzed by three dioxygenases: TDO2, predominantly expressed in the liver [14], and IDO enzymes (IDO1/IDO2) expressed in immune cells, kidneys, brain, placenta, and epididymis [15]. Essentially, these enzymes regulate Trp availability in the human body. The N-formyl-L-kynurenine is then converted into Kyn, a key metabolite that can be further processed into several bioactive downstream products. Kyn can be transaminated by kynurenine aminotransferases (KATs) to produce KYNA [16], a neuroprotective metabolite that acts as a competitive antagonist of glutamate receptors, such as N-methyl-D-aspartate (NMDA) receptors, and α7 nicotinic acetylcholine receptors (α7 nAChRs) [17]. Alternatively, Kyn can be hydroxylated by kynurenine monooxygenase (KMO) to form 3-HK, a metabolite capable of generating oxidative stress. 3-HK may be transaminated by KATSs to produce XA, another downstream metabolite implicated in immune regulation and cancer biology. Kynureninase (KYNU) then cleaves 3-HK to produce 3-HAA, which exerts complex biological effects and can act as either an antioxidant or pro-oxidant depending on the context [18]. The 3-HAA is subsequently oxidized by 3-hydroxyanthranilate 3,4-dioxygenase (3-HAO) to form QUIN, a potent NMDA receptor agonist and neurotoxin. The balance between KYNA and QUIN is critical for neuronal survival and function [17].

Beyond its metabolic role, the KYNP is central to immune regulation. Trp depletion and Kyn accumulation profoundly influence immune cell activation, tolerance, and inflammatory responses. The pathway contributes to cellular energy homeostasis through the generation of redox cofactors NAD^+^(P^+^)/NAD(P)H and supports immune tolerance, inflammation, and tumor progression [48]. Moreover, KYNP plays a critical role in maintaining immune and metabolic homeostasis by protecting immune-privileged tissues such as the eyes, brain, placenta, and colon from excessive inflammatory responses through the production of immunosuppressive intermediate metabolites [49]. The KYNP also contributes to tissue-specific regulation in many organs by generating metabolites with either neuroprotective (KYNA) or neurotoxic (QUIN) effects [50].

Unusual KYNP activation is observed across many tumor types and is increasingly recognized as a contributor to immune evasion and tumor progression. In cancer, enzymes like TDO2 and IDO1/IDO2 are frequently upregulated in tumor cells and tumor-associated immune cells. Their sustained activation can lead to local Trp depletion and accumulation of immunoregulatory metabolites of the KYNP, particularly Kyn. This metabolic reprogramming suppresses effector T-cell proliferation, promotes the differentiation of Tregs and creates an immunosuppressive TME, allowing cancer cells to escape immune surveillance and proliferate uncontrollably [22]. Beyond this immune modification, Kyn and its downstream metabolites can act as ligands and activators of the AhR, leading to pleotropic consequences. Following its activation, AhR translocates into the nucleus and regulates the expression of target genes involved in immunity, metabolism, and angiogenesis [23]. Furthermore, the IDO1-kyn-AhR axis upregulates PD-1 expression in CD8^+^ T-cells, providing an immune-tolerant microenvironment for tumor cells, triggering tumor development [24]. Moreover, elevated levels of KYNP metabolites such as QUIN and 3-HK contribute to oxidative stress and DNA damage, further supporting cancer development. In contrast, KYNA, acting as a neuroprotective metabolite, counteracts the excitotoxicity, a pathological process caused by excessive glutamate receptor activation that leads to neuronal injury and death, induced by QUIN, and functions as an endogenous antagonist of glutamate receptors, including the NMDA, α-amino-3-hydroxy-5-methyl-4-isoxazolepropionic acid (AMPA), and kainate receptors [25]. Through this antagonistic activity, KYNA helps maintain cellular redox balance and limits oxidative stress-induced DNA damage, demonstrating the pathway’s dual and specific role in cancer biology.

### 4.1. Phytochemical-Mediated Suppression of KYNP Entry Enzymes

Direct modulation of the rate-limiting enzymes of the KYNP, particularly IDO1 and, to a lesser extent, TDO2, emerged as one of the principal mechanisms through which phytochemicals interfere with Trp catabolism. Although the included studies employed diverse experimental approaches, ranging from enzymatic assays and cellular models to computational screening, they consistently identified IDO1 as the principal molecular target. This finding aligns with the reported function of IDO1 as the primary rate-limiting enzyme of the KYNP in many malignancies, where its activation increases immunological tolerance and accelerates tumor development, making it an appealing therapeutic target for cancer immunotherapy [51]. Other investigations further extended this concept by demonstrating that simultaneous inhibition of both IDO1 and TDO2 may provide a broader suppression of KYNP activity than selective inhibition of either enzyme alone.

Evidence from enzyme-based studies first demonstrated that several compounds can directly inhibit IDO1 catalytic activity. Anthraquinone derivatives isolated from Aloe exudates exhibited significant IDO1 inhibitory activity (IC50 = 39.41 to 53.93 µM), while kinetic analyses revealed an uncompetitive mode of inhibition, indicating direct interaction with the enzyme rather than indirect regulation of its expression [30]. Similarly, biochemical validation using fluorescence-based enzymatic assays has demonstrated that common dietary phytochemicals can directly suppress IDO1 catalytic activity. Although chemically unrelated to the Aloe-derived anthraquinone, flavonoids and phenolic acids including quercetin, luteolin, gallic acid, and phloretin produced concentration-dependent inhibition of IDO1 activity, in some cases achieving inhibitory potency comparable to reference small-molecule inhibitors [31]. Despite differences in chemical structure and experimental methodology, both studies consistently demonstrate that direct enzymatic inhibition of IDO1 represents a common mechanism shared by chemically diverse classes of phytochemicals.

In contrast to these enzyme-based investigations, Cao et al. (2023) explored the biological consequences of reduced IDO1 activity in vivo. Rather than directly inhibiting the enzyme, rosmarinic acid increased antitumor immunity and decreased tumor growth only in IDO1-silenced H22 hepatocellular carcinoma mouse models, showing that its therapeutic efficacy depended on earlier KYNP activity inhibition [32]. Compared to studies that demonstrated direct catalytic inhibition, these findings imply that phytochemicals may operate as biological amplifiers of IDO1 blocking, increasing T-cell responses and improving tumor suppression when KYNP activation has been reduced.

Although selective IDO1 inhibition predominated among the included studies, increasing evidence suggests that simultaneous inhibition of both IDO1 and TDO2 may provide a more comprehensive strategy for suppressing KYNP activation. The rationale for dual inhibition is further supported by findings that IDO1 and TDO2 might act as complementary enzymes in tumor Trp metabolism. As a result, selective inhibition of one enzyme may be compensated by the activity of another, allowing for continuous Kyn synthesis and the maintenance of an immunosuppressive TME [52]. Tanshinone-derived compounds originating from *Salvia miltiorrhiza* have emerged as potent dual inhibitors of IDO1 and TDO2, exhibiting submicromolar activity in enzymatic assays. Mechanistic studies revealed that IDO1 and TDO2 are inhibited through different molecular interactions, supporting the concept that simultaneous inhibition of both enzymes may prevent compensatory Kyn production [33]. These experimental findings were further supported by in silico screening approaches that have expanded the number of phytochemicals with predicted IDO1/TDO2 inhibitory potential. Computational docking identified naturally occurring alkaloids and polyphenol conjugates, such as chelirubine, hydroxy-sanguinarine, and epicatechin-5-O-glucuronide, as possible IDO1/TDO2 inhibitors, demonstrating stable interactions within the enzyme active sites, supported by favorable binding energies. Despite the absence of experimental validation, these findings support the feasibility of phytochemical-based dual-targeted methods [34].

Overall, existing evidence consistently points to direct regulation of IDO1 as the principal mechanism by which phytochemicals interfere with KYNP function. Nevertheless, an analysis of the research included shows clear progression in the field, from early studies focusing on selective enzymatic inhibition to more current techniques that include immunological regulation and dual IDO1/TDO2 targeting. While experimental validation of dual inhibitors is limited, the data indicates that multitarget inhibition of KYNP entry enzymes may be a more effective treatment approach than IDO1 inhibition alone.

### 4.2. Phytochemical-Mediated Regulation of Kynurenine Production and Downstream Metabolism

Beyond directly inhibiting the rate-limiting enzymes of the KYNP, some studies have investigated whether phytochemicals could influence the metabolic consequences of KYNP activation. Rather than focusing exclusively on IDO1 or TDO2 inhibition, these studies evaluated alterations in Kyn production and downstream metabolites, providing further insight into the functional impact of phytochemical-mediated KYNP modulation. Despite differences in phytochemical classes, cancer models, and experimental design, the available evidence consistently identified reduced Kyn production as the predominant metabolic outcome following KYNP modulation.

This observation was consistently demonstrated in studies targeting IDO1 activity through different mechanisms. A polyphenol-based, pH-responsive nanocarrier was developed by Li et al. for co-delivering docetaxel and the IDO1 inhibitor NLG919 to modulate KYNP activity in pancreatic ductal adenocarcinoma. Treatment significantly reduced Kyn levels and the Kyn/Trp ratio in tumor tissues, indicating effective suppression of IDO1-mediated Trp catabolism [35]. The Kyn/Trp ratio is generally recognized as a secondary biomarker for IDO1/TDO2 enzymatic activity and total KYNP activation, with higher values indicating greater Trp catabolism in cancer [53]. These metabolic alterations were accompanied by improved therapeutic efficacy, suggesting that restoration of Trp metabolism may contribute to enhanced antitumor responses [35]. Likewise, the natural alkaloid abrine was shown to directly bind IDO1, reduce IFN-γ-induced Trp catabolism, and decrease Kyn production in hepatocellular carcinoma models. In addition to its direct enzymatic effects, abrine inhibited transcriptional induction of IDO1 by suppressing the JAK1/STAT1 signaling axis and reducing aberrant m6A RNA methylation, thereby limiting IDO1 expression at multiple regulatory levels. This coordinated regulation resulted in reduced Kyn production, demonstrating that phytochemicals can modulate KYNP metabolism through both enzymatic and transcriptional mechanisms [36]. Although the molecular mechanisms differed substantially between the two studies, both consistently demonstrated that suppression of IDO1 activity ultimately converges on reduced Kyn production, supporting Kyn as a robust metabolic indicator of successful KYNP inhibition.

In contrast to studies primarily evaluating Kyn levels, other investigations focused on downstream metabolites of the pathway. A chemical modification of KYNA isolated from *Castanea sativa* L. produced derivatives, including the alkaloid 3-(2′-pyrrolidinyl)-kynurenic acid lactam (3-PKA-L), which was evaluated relative to KYNA. The 3-PKA-L selectively induced significant cytotoxicity through activation of the extrinsic apoptotic pathway, accompanied by morphological alterations and reduced colony formation in Castration-resistant prostate cancer (CRPC) cell lines [37]. These findings suggest that structural modifications of Kyn-related compounds may substantially alter their biological activity, highlighting naturally occurring KYNA derivatives as promising candidates for future anticancer drug development.

Overall, the included studies demonstrate that phytochemicals can modulate Kyn production and downstream metabolism, underscoring the importance of metabolic control in KYNP-targeted treatments. However, these metabolic changes are rarely observed in isolation and are typically accompanied by broader alterations in oncogenic signaling networks. The complementary molecular mechanisms are discussed in the following section.

### 4.3. Cellular Consequences of Phytochemical-Mediated KYNP Modulation in Cancer

While the previous section demonstrated that phytochemicals consistently alter the metabolic output of the KYNP, several studies further investigated whether these metabolic changes are accompanied by broader alterations in cancer cell behavior. Rather than investigating KYNP metabolism as an isolated process, the included studies collectively show that modulation of KYNP-associated parameters occurs alongside alterations to oxidative stress, intracellular signaling, extracellular matrix remodeling, cell migration, and treatment responsiveness. Although the extent of KYNP research varied greatly between studies, all found coordinated cellular responses that contribute to the overall anticancer action of phytochemicals.

Among the included studies, rutin provided one of the most comprehensive examples of the cellular consequences associated with KYNP modulation in cancer. Using both two-dimensional monolayer cultures and three-dimensional glioblastoma spheroids, rutin modulated the IDO1-Kyn axis in a context-dependent manner. In stable spheroids, treatment was associated with increased extracellular L-kynurenine levels and IDO1 expression, whereas under chemosensitization conditions, rutin reduced Kyn levels when administered alone, indicating that its effects on KYNP depend on the experimental setting. Beyond its effects on KYNP, rutin attenuated intracellular ROS accumulation, suppressed IL-6/STAT3 and PI3K/AKT signaling, reduced nitric oxide production, and decreased the expression of extracellular matrix proteins involved in tumor invasion, including fibronectin, laminin, and MMP2. These combined molecular alterations were associated with decreased glioblastoma cell migration, breakdown of spheroid organization, and greater sensitivity to temozolomide [38]. These findings are consistent with earlier research demonstrating that oxidative stress and inflammatory signaling are strongly linked to IDO1 activity and can impact KYNP activation in the TME [54]. Furthermore, previous evidence has shown that IL-6/STAT3 signaling directly maintains IDO1 expression and Kyn synthesis in cancer cells, implying that inhibiting STAT3 activity may lead to lower KYNP activation [55]. Unlike the research addressed in the previous section, which largely investigated metabolic outcomes, this study shows that modification of KYNP-associated parameters can occur concurrently with broad control of cellular pathways implicated in tumor growth and chemoresistance.

A similar relationship between Kyn metabolism and cellular responses was observed in studies investigating *Bersama abyssinica* and industrial hemp (*Cannabis sativa *L.) [39,40], although these studies examined different biological endpoints. In contrast to the investigation of rutin, which examined many signaling pathways at the same time, both investigations concentrated on the link between Kyn metabolism, oxidative balance, inflammation, and cell proliferation. *Bersama abyssinica* extracts exhibited significant antioxidant and antiproliferative activity in HCT116 colon cancer cells, while preventing serotonin-induced depletion of KYNA, suggesting that KYNP metabolite homeostasis is maintained under situations of increased tumor cell proliferation [39]. Similarly, water extracts of industrial hemp (*Cannabis sativa* L.) were evaluated using both HCT116 colon cancer cells and an ex vivo lipopolysaccharide (LPS)-induced inflammatory model using isolated rat colon and liver tissues. Treatment lowered HCT116 cell viability, attenuated LPS-induced alterations in serotonin, dopamine, and Kyn turnover in rat colon tissue, and suppressed prostaglandin E2 (PGE2) synthesis in both colon and liver specimens [40]. This observation is consistent with evidence showing that PGE2 produced by cyclooxygenase-2 (COX-2)-expressing neutrophils promotes IDO1 upregulation in cancer cells, suggesting that suppression of PGE2 may indirectly attenuate KYNP activation [56]. Specifically, treatment reduced the pro-inflammatory 3-HK/KYNA ratio, while increasing KYNA levels, indicating a shift towards a less inflammatory KYNP profile. Although bioinformatics analysis predicted a potential interaction between a constituent of the extracts, carvacrol, and IDO1, there was no experimental validation [40].

A complementary perspective was provided by the study of *Ephedra foemina*, which approached the KYNP from a different angle. Rather than investigating modulation of KYNP activity, the authors identified 6-methoxykynurenic acid (6-mKYNA), a naturally occurring derivative of KYNA. Initial in vivo evaluation using Ehrlich ascites carcinoma-bearing mice displayed strong antiproliferative efficacy and considerable reductions in the tumor biomarkers CA-19.9 and CA-125. Subsequent in vitro experiments in MCF-7 breast cancer cells demonstrated that isolated 6-mKYNA exhibited the strongest cytotoxic activity among all purified compounds and further reduced CA-19.9 and CA-125 in a dose-and time-dependent manner [41]. Unlike the previous studies, which examined phytochemical-mediated regulation of Kyn metabolism, this work highlights the intrinsic biological activity of a KYNP-derived metabolite, suggesting that downstream metabolites of the pathway may themselves represent promising anticancer agents.

Collectively, the available evidence suggests that phytochemical-mediated modulation of KYNP goes beyond changes in Trp metabolism and is consistently accompanied by broader cellular alterations related to oxidative stress, inflammatory signaling, extracellular matrix remodeling, migration, proliferation, and therapeutic response. Although the included studies focused on different phytochemicals and experimental models, they all agreed that KYNP regulation is part of a coordinated system of cellular responses that contribute to natural compounds’ overall anticancer efficacy. The effects of these findings on tumor immune regulation are discussed in the next section.

### 4.4. Phytochemical-Mediated KYNP Regulation of Antitumor Immunity

Beyond their direct impact on Kyn metabolism and cancer cell biology, multiple studies have shown that phytochemicals have important immunomodulatory effects by regulating the KYNP. This is consistent with the known role of aberrant KYNP activation in establishing tumor immunity by suppressing effective anticancer immune responses [54]. Even though various phytochemicals targeted different components of the pathway, all studies repeatedly found that suppressing KYNP activity was associated with restoration of antitumor immunity, emphasizing immune regulation as one of the primary biological consequences of phytochemical-mediated KYNP modulation. Two principal therapeutic strategies emerged from the available evidence: (i) inhibition of IDO1-dependent Kyn production and (ii) blockade of downstream AhR signaling.

The first strategy was especially noticeable in research focusing on IDO1-dependent Kyn synthesis. Liang et al. (2023) demonstrated that the natural alkaloid abrine directly binds to IDO1, reduces IFN-γ-induced Trp catabolism, and decreases Kyn production in hepatocellular carcinoma models. Functionally, inhibition of Kyn accumulation led to reduced expression of immune checkpoint molecules Programmed Death Ligand 1 (PD-L1) and CD47, enhanced macrophage phagocytosis, increased infiltration of CD8^+^T cells, and decreased Foxp3^+^ Tregs within tumor tissues [36]. Importantly, abrine improved the efficacy of anti-PD-1 immunotherapy in vivo, in Hepa1-6 xenograft mice, underscoring the translational relevance of upstream KYNP inhibition as a strategy to enhance immune checkpoint blockade [36]. Similarly, Li et al. (2025) reported that suppression of Kyn production using polyphenol-based, pH-responsive nanocarrier co-delivering docetaxel and the IDO1 inhibitor NLG919 resulted in increased CD8^+^ T-cell infiltration, enhanced dendritic cell maturation, and reduced Foxp3^+^ Tregs recruitment in vivo, and marked inhibition of pancreatic tumor growth [35]. Despite using distinct treatment techniques, both studies consistently show that suppression of IDO1 activity and Kyn production alters the tumor immunological context, leading to a more effective antitumor immune response.

Comparable immunological effects were also observed in the treatment of a H22 hepatocellular carcinoma xenograft model with rosmarinic acid and IDO1-shRNA. In contrast with abrine, which directly inhibits IDO1 activity, this strategy, the combination of a natural compound with genetic silencing of the IDO1 gene, increased the CD4^+^/CD8^+^ T-cell ratio, reduced the apoptosis of CD8^+^ T-cells, decreased the Foxp3+ Tregs populations, and elevated the levels of IFN-γ and Interleukin-2 (IL-2), while reducing the immunosuppressive cytokines Interleukin-10 (IL-10) and Tumor Necrosis Factor α (TNF-α) [32]. This evidence further supports the concept that targeting IDO1 can reverse cancer-related immunosuppression and enhance antitumor immunity.

Similar immunological results were obtained with the traditional herbal formulation Feiji Recipe, but via a more advanced multi-component intervention. While abrine and the polyphenol-based nanocarrier predominantly modulated immune responses by inhibiting IDO1-dependent Kyn synthesis, Feiji Recipe primarily reduced IDO1 expression in vivo, prolonging survival in an orthotopic lung cancer mouse model while significantly decreasing CD4^+^CD25^+^ and FoxP3^+^ Tregs populations [42]. Although there are variations in phytochemical composition and experimental design, all three studies repeatedly show that inhibiting IDO-mediated immune tolerance restores T-cell function and prevents tumor immune escape, highlighting IDO1 as a key immunometabolic target for phytochemical-based cancer therapy.

Another study of Chen et al. (2022) investigating Myricetin, a naturally occurring flavonoid, in human lung cancer cell lines (A549, NCI-H1650, and NCI-H460), showed that it markedly suppressed IFN-γ-induced IDO1 and PD-L1 expression at both mRNA and protein levels, resulting in reduced extracellular Kyn production through inhibition of the JAK/STAT/IRF1 signaling axis. Myricetin restored the activity of the T-cells in co-culture, as proved by increasing proliferation, survival, CD69^+^ expression and IL-2 secretion of Jurkat-PD-1 T-cells, which were previously suppressed by IFN-γ-treated lung cancer cells. Mechanistically, IFN-γ upregulated PD-L1 and IDO1 at the transcriptional level via the JAK-STAT-IRF1 axis, which myricetin targeted and inhibited [43].

Unlike myricetin and abrine, which primarily suppress IFN-γ-induced IDO1 expression through inhibition of the JAK/STAT signaling, resveratrol regulated IDO1 through upregulation of connexin 43, revealing an alternative upstream mechanism of controlling KYNP activity. Resveratrol administered alone or in combination with *Salmonella enterica* serovar Choleraesuis was evaluated in the murine melanoma cell line B16F10 to investigate the relationship between connexin 43 (Cx43) and IDO1-mediated immune regulation. Treatment significantly increased Cx43 expression while reducing IDO1 protein expression and Kyn production. Moreover, silencing Cx43 reversed the inhibitory effects of both treatments on IDO1, indicating that Cx43 acts as an upstream regulator of IDO1 expression. As a result, inhibiting IDO1 activity reduced IDO1-mediated T-cell suppression, indicating that anticancer immune responses could be restored. These findings reveal that Cx43 as unique mediator that links phytochemical therapy to inhibition of KYNP-driven immunity [44].

While previous studies focused on restricting Kyn synthesis, more recent research has shown that immunological restoration may also be accomplished by inhibiting downstream KYNP signaling via AhR. Icariside I, a prenylflavonoid isolated from *Epimedium*, was identified as a novel inhibitor of the IDO1-Kyn-AhR signaling axis. Molecular docking showed strong binding of icariside I to IDO1, while in vivo, and in vitro experiments, in melanoma cells (B16F10), demonstrated significant reductions in Kynand its downstream metabolites KYNA, and XA levels, as well as the expression of key regulatory components of the pathway, including IDO1, KAT, KMO and AhR. Suppression of the IDO1-Kyn-AhR-p27 cascade restored JAK/STAT1 signaling, promoted cancer cell apoptosis, enhanced CD8+ T-celll infiltration and CD8^+^/CD4^+^ ratio, and improved antitumor immunity, thus overcoming the tumor immune escape [45].

Using B16F10 melanoma-bearing mice together with complementary in vitro experiments, Li et al. (2024) used a similar technique, identifying natural AhR antagonists in *Salvia miltiorrhiza* and synthesizing optimized analogues with better biological activity. These compounds inhibited AhR signaling, suppressed PD-1/PD-L1 expression, increased CD8^+^ T-cell proliferation and IFN-secretion, reducing tumor-associated macrophage infiltration, and significantly inhibiting tumor growth in vivo. Unlike icariside I, which inhibits the upstream synthesis of endogenous AhR ligands, Salvia-derived compounds operate directly at the receptor level [46]. Nonetheless, both investigations consistently show that pharmacologically inhibiting the Kyn-AhR axis efficiently reverses tumor-associated immune suppression and boosts anticancer immunity.

In conclusion, the results show that phytochemicals restore anticancer immunity via modulating the KYNP. Although some compounds predominantly inhibit IDO1-dependent Kyn synthesis, others disrupt downstream AhR signaling, resulting in the restoration of cytotoxic T-cell function, decrease in regulatory immunological populations, and increase in anticancer immune responses. Despite using different phytochemicals, cancer models, and molecular targets, the existing data consistently support KYNP as a major immunometabolic regulator, whose modulation represents a promising technique for enhancing phytochemical-based cancer immunotherapy.

### 4.5. Methodological Quality and Implications for Interpretation

The design-specific appraisal tempers the strength of the mechanistic conclusions. In animal studies, sparse reporting of sequence generation, allocation concealment, random housing, and blinding made most domains unclear; unexplained outcome-specific sample-size differences in [35,45] further reduced confidence. In vitro evidence was generally better documented, especially where independent replication, orthogonal target/functional assays, vehicle controls, and non-tumour comparators were used [38,43]. Nevertheless, several studies incompletely reported compound purity or solvent exposure, biological versus technical replication, data exclusions, or correction for multiple comparisons. Direct enzyme inhibition therefore supports target engagement but does not, by itself, establish antitumour efficacy.

These limitations are particularly important for studies [31,34,41,46], for which cancer relevance, computational reproducibility, internal consistency, or unexplained outcome-specific sampling constrained confidence. The body of evidence should therefore be viewed as hypothesis-generating. Future studies should use prospectively specified protocols, randomization and blinded outcome assessment for animal experiments, justified sample sizes, independent biological replicates, appropriate vehicle and positive controls, exposure justification, validated KYNP readouts, and open sharing of analysis inputs and code.

### 4.6. Summary of Phytochemical-Mediated KYNP Regulation

Overall, the included studies indicate that phytochemicals can modulate the KYNP at multiple interconnected levels. Several compounds directly target the KYNP entry enzymes IDO1 and TDO2, thereby influencing Trp catabolism and Kyn production, whereas others affect downstream Kyn metabolites and metabolic balance. These molecular effects are associated with diverse cellular consequences, including inhibition of cancer cell proliferation and migration, induction of apoptosis, and enhanced sensitivity to anticancer treatment. In addition, phytochemically mediated KYNP modulation can influence tumor-associated immune responses through pathways such as the Kyn-AhR axis, PD-1/PD-L1 signaling, and T-cell activity. Collectively, these findings highlight the potential of phytochemicals to regulate the KYNP through interconnected metabolic, cellular, and immune mechanisms in cancer.

## 5. Conclusions

This systematic review highlights the increasing potential of phytochemicals as modulators of the KYNP in cancer. Although most studies focused on suppressing IDO1 and lowering Kyn levels, current research reveals that phytochemicals can also influence downstream signaling pathways such as AhR activation, immune cell function, and the TME. These data suggest that targeting the KYNP might be an alternative strategy for restoring antitumor immunity and improving the efficacy of current cancer treatments. However, there are some limitations. The majority of the current data is based on in vitro investigations or animal models, with clinical trials still missing. Furthermore, the included studies show significant variation regarding the use of cancer models, phytochemical sources, experimental methods, and outcome measures, making direct comparison challenging. Another significant restriction is that the vast majority of the investigations are focused solely on IDO1, whereas the impact of IDO2 remains mostly unknown. None of the included studies evaluated phytochemical-mediated modulation of IDO2, showing a significant gap in the current literature. Future research should focus on validating these findings in preclinical and clinical studies, while extending the research to additional components of the KYNP, specifically IDO2. Overall, phytochemicals are promising candidates for the development of innovative anticancer treatments that target KYNP, but more data is needed before their clinical relevance can be validated.

## Figures and Tables

**Table 1:** Representative phytochemicals with well-established biological activities. Chemical structures were obtained from PubChem Compound records (National Center for Biotechnology Information, NCBI).

Phytochemical	Chemical Structure	Established Biological Activity
Quercetin	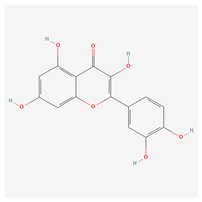	Antioxidant activity; ROS scavenging; inhibition of lipid peroxidation
Epigallocatechin gallate	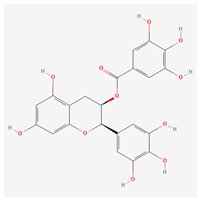	Antioxidant activity; ROS scavenging; inhibition of lipid peroxidation
Caffeic acid	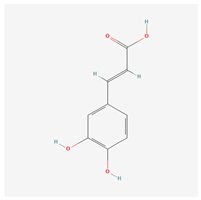	Strong antioxidant activity; free-radical stabilization
Ferulic acid	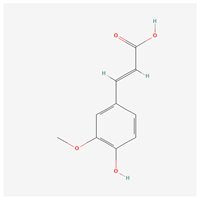	Strong antioxidant activity; free-radical stabilization
Lycopene	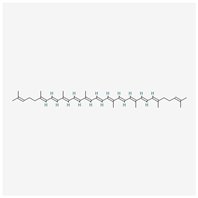	Reduction in oxidative damage to lipids, proteins, and DNA
Lutein	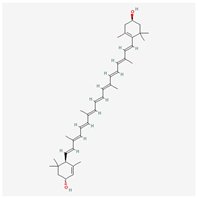	Reduction in oxidative damage to lipids, proteins, and DNA

**Table 2:** Inclusion and exclusion criteria for article selection.

Criteria Category	Inclusion Criteria	Exclusion Criteria
Additional keywords in filter selection of Scopus database	Kynurenine, Plant Extract, Polyphenols, Tumor growth, Cancer Therapy, Cell Viability, Cell Proliferation, Drug Effect, Flavonoids, Indoleamine 2,3 Dioxygenase, Kynurenic Acid, Phytochemical, Plant Extracts, Polyphenol, Quinolinic Acid, Tryptophan Metabolism	Papers that did not include any of the search terms, or where the keywords were unrelated to the kynurenine pathway, phytochemicals or cancer.
Subject Areas	Biochemistry, Genetics and Molecular Biology, Pharmacology, Toxicology and Pharmaceutics, Medicine, Chemistry, Immunology and Microbiology, Neuroscience, Agricultural and Biological Sciences.	Computer Science, Chemical Engineering, Environmental Science.

**Table 3:** Summary of the included studies investigating phytochemical targeting KYNP in cancer.

No.	Phytochemical	Mechanistic Category	Experimental Model	KYNP Target	Main Findings	Reference
1	Anthraquinone derivatives isolated from Aloe exudates	Direct modulation of KYNP entry enzymes	Enzyme kinetic analysis	IDO1	Anthraquinone derivatives exhibited significant IDO1 inhibitory activity (IC_50_ = 39.41–53.93 μM).	[30]
2	Quercetin, luteolin, gallic acid, and phloretin		Enzyme kinetic analysis	IDO1	Identified natural products with significant IDO1 inhibitory activity.	[31]
3	Rosmarinic acid		H22 hepatocellular carcinoma mouse model with IDO1 silenced	IDO1	Rosmarinic acid enhanced the effects of IDO1 silencing, leading to stronger inhibition of tumor cell growth and increased antitumor activity.	[32]
4	Tanshinone-derived compounds		Enzyme kinetic analysis	IDO1/TDO2	Derivatives of natural tanshinones acted as potent dual IDO1/TDO2 inhibitors, highlighting simultaneous targeting of both entry enzymes of the KYNP.	[33]
5	Chelirubine, hydroxy-sanguinarine, and epicatechin-5-O-glucuronide		In silico	IDO1/TDO2	Computational analyses identified several phytochemicals with favorable binding affinities toward both IDO1 and TDO2, supporting their potential as dual KYNP inhibitors.	[34]
6	Polyphenol-based nanoparticles co-loaded with NLG919 and docetaxel	Regulation of Kynurenine production or downstream metabolites/KYNP-driven immunomodulation	Pancreatic ductal adenocarcinoma cell lines	IDO1, Kyn	Polyphenol-based nanoparticles enhanced chemo-immunotherapy by reducing IDO1-mediated Kyn production, promoting antitumor immunity, and suppressing tumor growth.	[35]
7	Abrine		Hepatocellular carcinoma cell lines and tumor xenografts	IDO1, Kyn, Trp	Abrine inhibited IDO1 activity, reduced Kyn production, restored T-cell function, and significantly enhanced anti-PD-1 immunotherapy.	[36]
8	Alkaloid 3-PKA-L isolated from *Castanea sativa *L.	Regulation of Kynurenine production or downstream metabolites	Castration-resistant prostate cancer cell lines	KYNA	3-PKA-L reduced cell viability, inhibited colony formation, and induced extrinsic apoptosis	[37]
9	Rutin	Cellular Consequences Associated with KYNP Modulation in Cancer	2D-3D Glioblastoma spheroids	IDO1, Kyn	Rutin enhanced temozolomide sensitivity, reduced chemoresistance, modulated IDO1/KYNP signaling, and decreased migration of glioblastoma spheroids.	[38]
10	*Bersama abyssinica*		HCT116 colon cancer cells	KYNA	Extracts exhibited antioxidant and antiproliferative effects in HCT116 cells and prevented serotonin-induced KYNA depletion.	[39]
11	Industrial hemp (*Cannabis sativa*)		HCT116 colon cancer cells and mouse model	3-HK/KYNA ratio KYNA	Water extracts reduced HCT116 cell viability, decreased 3-HK/KYNA ratio, increased KYNA, and reduced PGE2 levels.	[40]
12	*Ephedra foemina*		Ehrlich ascites carcinoma-bearing mice, MCF-7 BC cells	6-mKYNA derivative of KYNA	6-mKYNA exhibited potent antiproliferative activity and reduced CA-19.9 and CA-125 levels.	[41]
13	Feiji Recipe	KYNP-driven immunomodulation	Orthotopic lung cancer mouse model	IDO1	Feiji Recipe reduced IDO1 expression, suppressed Tregs, and prolonged survival.	[42]
14	Myricetin		Lung cancer cell lines (A549, NCI-H1650, and NCI-H460)	IDO1, Kyn	Myricetin suppressed IFN-γ-induced IDO1, PD-L1, and Kyn production via the JAK/STAT/IRF1 pathway, restoring T-cell activity.	[43]
15	Resveratrol		Murine melanoma cell line (B16F10)	IDO1	Resveratrol upregulated Cx43, reduced IDO1 expression and Kyn production, and restored T-cell-mediated antitumor immunity.	[44]
16	Icariside I		Murine melanoma model (B16F10)	IDO1, Kyn, KYNA, XA, KAT, KMO, AhR	Icariside I inhibited IDO1-Kyn-AhR signaling axis, restored JAK1/STAT1 signaling, enhanced CD8^+^ T-cell immunity, and promoted tumor cell apoptosis.	[45]
17	*Salvia miltiorrhiza*		Murine melanoma model (B16F10)	AhR	Extracts directly antagonized AhR, suppressed PD-1/PD-L1 expression, enhanced CD8^+^ T-cell immunity, and reduced tumor growth.	[46]

Abbreviations: KYNP, kynurenine pathway; IDO1, indoleamine 2,3-dioxygenase 1; TDO2, tryptophan 2,3-dioxygenase; Trp, tryptophan; Kyn, kynurenine; KYNA, kynurenic acid; PD-1, Programmed Death 1; PD-L1, Programmed Death Ligand 1; 3-PKA-L, 3-(2′-pyrrolidinyl)-kynurenic acid lactam; 3-HK, 3-hydroxykynurenine; BC, breast cancer; 6-mKYNA, 6-methoxykynurenic acid; Tregs, regulatory T-cells; XA, xanthurenic acid; KAT, kynurenine aminotransferase; KMO, kynurenine monooxygenase; AhR, aryl hydrocarbon receptor; Cx43, connexin 43; PGE2, prostaglandin E2; IFN-γ, interferon-γ.

**Table 4:** Study-level methodological quality and risk-of-bias synthesis.

Design/Tool	Confidence	Primary Methodological Consideration	Study
Enzyme; SciRAP	Moderate	Replicated human IDO1 assay; incomplete compound characterization and indirect cancer relevance.	[30]
Assay/in silico; SciRAP	Limited/indirect	Crude lens-homogenate system and indirect cancer relevance.	[31]
Animal; SYRCLE	Unclear RoB	Randomization stated without method; concealment, housing, and blinding unreported.	[32]
Enzyme/cell; SciRAP	Moderate	Orthogonal characterization; high cellular DMSO and incomplete statistical detail.	[33]
In silico; reproducibility	Moderate-low	Methods/software reported; no independent trajectories, convergence testing, reusable inputs/code, or experimental validation.	[34]
Cell/animal; both	Moderate	Broad controls; animal safeguards unreported and outcome sample sizes varied.	[35]
Cell/animal; both	Moderate	Orthogonal evidence; animal randomization method and blinding unreported.	[36]
Cell; SciRAP	Moderate	Replicated endpoints and normal comparator; high concentration and incomplete control coverage.	[37]
2D/3D cell; SciRAP	Moderate-high	Strong replication/statistics; passage and mycoplasma status not reported.	[38]
Cell; SciRAP	Moderate	Profiled extract and replicated assays; single cancer-test concentration.	[39]
Cell/ex vivo; SciRAP	Moderate	Characterized extract and power calculation; some cell methods reported by citation.	[40]
Cell/animal; both	Limited	Conflicting exposure/implantation details and weak multi-group statistics.	[41]
Cell/animal; both	Moderate-low	Relevant model; formula standardization and animal methods incomplete.	[42]
Cell; SciRAP	Moderate-high	Multiple lines and independent replication; passage/mycoplasma and post-hoc details absent.	[43]
Cell; SciRAP	Moderate	Replicated genetic/cell evidence; multiple simple t-tests and no in-vivo experiment.	[44]
Cell/animal; both	Moderate	Randomized tumour model; no sequence method/blinding and unexplained sample-size variation.	[45]
Cell/animal; both	Moderate	Randomization stated without method/blinding; immune outcomes used *n* = 5 from *n* = 7 groups without explanation.	[46]

Abbreviations: RoB, risk of bias. Confidence labels are narrative syntheses, not validated composite scores. Domain-level ratings and evidence quotations are provided in the Supplementary File.

## Data Availability

No new data were created or analyzed in this study. Data sharing is not applicable to this article.

## Supplementary Materials

The following supporting information can be downloaded at: https://www.mdpi.com/article/10.3390/ijms27177512/s1.

## Abbreviations

The following abbreviations are used in this manuscript:

KYNP	Kynurenine pathway
IDO1	Indoleamine 2,3-dioxygenase 1
IDO2	Indoleamine 2,3-dioxygenase 2
TDO2	Tryptophan 2,3-dioxygenase
UV	Ultraviolet
EGCG	Epigallocatechin gallate
ROS	Reactive oxygen species
Trp	Tryptophan
Kyn	Kynurenine
KYNA	Kynurenic acid
QUIN	Quinolinic acid
KATs	Kynurenine aminotransferases
KMO	Kynurenine monooxygenase
3-HK	3-Hydroxykynurenine
XA	Xanthurenic acid
KYNU	Kynureninase
3-HAA	3-hydroxyanthranilic acid
3-HAO	3-hydroxyanthranilate 3,4-dioxygenase
α7 nAChRs	α7 nicotinic acetylcholine receptors
TME	Tumor microenvironment
Tregs	Regulatory T-cells
AhR	Aryl hydrocarbon receptor
3-PKA-L	3-(2′-pyrrolidinyl)-kynurenic acid lactam
CRPC	Castration-resistant prostate cancer
LPS	Lipopolysaccharide
PGE2	Prostaglandin E2
6-mKYNA	6-methoxykynurenic acid
PD-1	Programmed Death 1
PD-L1	Programmed Death Ligand 1
IL-2	Interleukin 2
IL-10	Interleukin 10
TNF-α	Tumor Necrosis Factor Alpha
Cx43	Connexin 43
NMDA	N-methyl-D-aspartate
NF-κB	Nuclear Factor kappa-light-chain-enhancer of activated B cells
AMPA	α-amino-3-hydroxy-5-methyl-4-isoxazolepropionic acid
IFN-γ	Interferon-γ
COX-2	Cyclooxygenase-2
